# Detection and evolutionary analysis of picobirnaviruses in treated wastewater

**DOI:** 10.1111/1751-7915.12239

**Published:** 2014-12-27

**Authors:** Shiwei Zhang, Ru Bai, Run Feng, Hongxun Zhang, Lixin Liu

**Affiliations:** 1 College of Life Science, University of Chinese Academy of SciencesNo.19A, Yuquan Road, Shijingshan District, Beijing, 100049, China; 2 Key Laboratory for Polymeric Composite and Functional Materials of Ministry of Education, School of Chemistry and Chemical Engineering, Sun Yat-Sen UniversityNo. 135, Xingang Xi Road, Guangzhou, 510275, China; 3 State Environmental Protection Key Laboratory of Microorganism Application and Risk Control (SMARC), Tsinghua UniversityBeijing, 100084, China

## Abstract

Wastewater contains numerous viruses. In this study, picobirnaviruses (PBVs) were detected in the stream of a wastewater treatment plant in Changsha, Hunan province, China, and evolutionary analysis of the isolated PBVs was performed. The phylogenetic tree revealed that the PBVs were highly divergent and could be classified into six distinct groups according to their hosts. Among these groups, pairwise comparison of the six groups revealed that the nucleotide distance of group 4 (bootstrap value = 0.92; nucleotide identity = 94%) was the largest. Thus, group 4 might represent a new division of PBVs. Comprehensive analysis of the obtained PBV sequences to investigate their evolutionary history and phylodynamics revealed that group 5 (PBVs from monkey) exhibited maximum polymorphism (K = 30.582, S = 74, η = 98, Pa = 47) and lowest nucleotide substitutions per site per year (6.54E-3 subs per site per year), except group 4. Maximum clade credibility tree indicated that group 5 appeared earlier than the other groups. In conclusion, this study detected PBVs in treated wastewater in China, and identified a new PBV group. Furthermore, among these PBVs, group 5 was found to survive longer and present a balance between PBVs and their monkey host.

## Introduction

Sewage or wastewater may contain numerous viruses disseminated from the hosts and provides an ideal environment for the growth of diverse viruses and their host species (Lodder and de Roda Husman, 2005; Cantalupo *et al*., 2011). The viruses that exist in the wastewater may come into contact with humans through many ways and, hence, are serious potential threats to human health. Recently, many studies have been carried out to examine the viruses in sewages (van den Berg *et al*., 2005; Rosario *et al*., 2009; Symonds *et al*., 2009). The development of sequencing technique, especially the metagenomic method, has provided a great opportunity to gain a better understanding of viruses (Thurber *et al*., 2009). However, the vast majority of the sequences of viruses are difficult to read or not related to known viruses, and thus could not be studied (Breitbart *et al*., 2003).

In the present study, picobirnaviruses (PBVs) were detected in the stream of a wastewater treatment plant in China, and evolutionary analysis of the isolated PBVs was carried out. PBVs widely exist in wastewater and have been classified into *Picobirnaviridae* family by the International Committee of Taxonomy on Viruses. They are small (Φ 35–40 nm), non-enveloped, dsRNA viruses with a bisegmented genome (Ganesh *et al*., 2012; Mondal *et al*., 2013). The large genomic segment or segment 1 is 2.2–2.7 kb and encodes the capsid protein. The small segment or segment 2 is 1.2–1.9 kb and encodes the RNA-dependent RNA polymerase (RdRp). Based on the sequences of this gene, PBVs have been classified into two genogroups represented by the Chinese strain 1-CHN-97 (AF246939, prototype of genogroup I) and US strain 4-GA-91 (AF246940, prototype of genogroup II) (Rosen *et al*., 2000; Bhattacharya *et al*., 2007).

PBVs widely exist in the environment and have a wide range of hosts, including humans (Smits *et al*., 2012), rabbits (Ludert *et al*., 1995; Green *et al*., 1999), pigs (Martínez *et al*., 2010; Smits *et al*., 2011), cattle (Ghosh *et al*., 2009), horses (Ganesh *et al*., 2011), birds (Chandra, 1997), monkeys (Wang *et al*., 2007; 2012) and foxes (Bodewes *et al*., 2013). Currently, many studies have been carried out to analyse PBVs at the epidemiologic and phylogenetic levels. Although phylogenetic tree is the most valuable and common technique of computational analysis of viral evolutionary changes, additional methods are required for detailed investigation of the evolution and phylodynamics of PBVs to gain in-depth understanding of how PBVs transmitted among a wide range of hosts (Nates *et al*., 2011). In the present study, phylogenetic tree was used to analyse the diversity of PBVs, and five comparative techniques, namely phylogenetic analysis, polymorphism analyses, inference of selection pressures acting on PBVs, measurement of evolutionary rate and construction of maximum clade credibility (MCC) tree, were employed to exhaustively study the evolutionary process of PBVs isolated from the stream of a wastewater treatment plant in China.

## Results

### Detection and sequencing of PBVs

**Fig 1:**
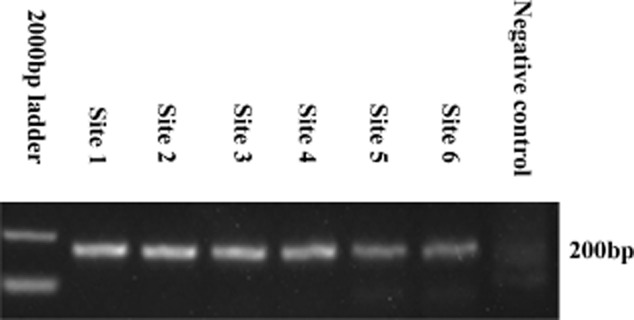
Agarose gel electrophoresis showing amplified products of 200 bases in 40 cycles of amplification by using PBVs primers. M: molecular weight standard marker, site 1–6: PCR product of six sites from treated water plant in Changsha Hunan province (China) with the amplicons length of 200 bp, N: negative control.

The Polymerase Chain Reaction (PCR) method was used to detect PBVs with partial RdRp gene (200 bp) from the six samples of treated wastewater. As shown in Fig. 1, for all the six samples, a distinctive 200 bp band could be detected in agarose gel (2%) with ethidium bromide under UV light. To better understand the diversity of the PBVs in treated wastewater, a total of 300 (∼ 50 products per sample) cloned PBV PCR products (∼ 200 bp) of the RdRp gene were sequenced by Sangon (Sangon, Beijing, China). After removing the error and similar identity sequences by blast analysis, 139 sequences (Table S1) were obtained and deposited in GenBank under accession numbers KJ135791 to KJ135929.

### Phylogenetic analysis

To examine the genetic relatedness between the PBVs from treated wastewater and known PBVs, the top three similar known sequences from the National Center for Biotechnology Information (NCBI) corresponding to the 139 sequences were chosen as reference sequences to construct the phylogenetic tree. A total of 65 known sequences (Table S2) were obtained from the NCBI database by using blast analysis. These 65 sequences included monkey strains (16 sequences), avian strains (11 sequences), porcine strains (9 sequences), human strains (7 sequences), fox strain (1 sequence) and uncultured strains (21 sequences). Thus, a total of 204 sequences were used to construct the phylogenetic tree by using mega 5.0.

**Fig 2:**
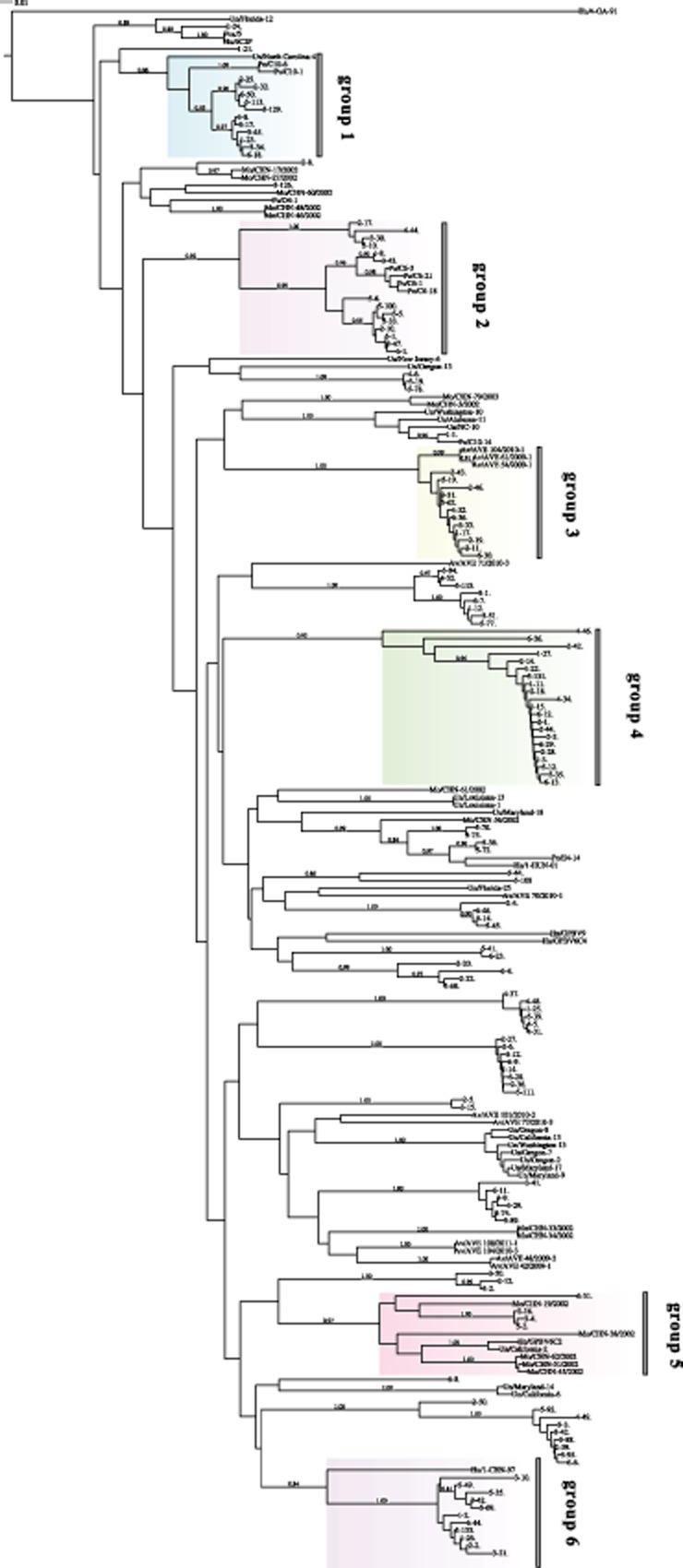
Neighbour-joining (Kimura 2-parameter model) phylogenetic tree of ∼ 200 bp segment 2 of the PBVs (genotype I) RNA-dependent RNA polymerase gene. A total of 204 sequences (139 sequences we obtained and 65 referenced sequences) were used to construct phylogenetic tree by mega 5.0. Six groups were displayed by different colours. A bootstrap value > 0.8 was indicated for the corresponding nodes, and the tree was statistically supported by bootstrapping with 1000 replicates. Bar, 0.01 substitutions per nucleotide.

From the constructed phylogenetic tree (Fig. 2), it could be observed that the PBVs from the treated wastewater had a high genetic diversity and that all the sequences had nucleotide identities ranging between 42% and 100%. With regard to the reliability of data analysis, sequences with bootstrap values > 0.8 were categorized as one group. Thus, of the total 139 sequences, 53 sequences (Table S3) were grouped with known host sequences: 26 sequences were grouped with porcine PBVs (average nucleotide identity = 76%; owing to the relatively low nucleotide identity, it was hypothesized that there were two different porcine PBVs); 12 sequences were grouped with avian PBVs (average nucleotide identity = 98%); 11 sequences were grouped with human PBVs (average nucleotide identity = 96%); and four sequences were grouped with monkey PBVs (average nucleotide identity = 81%) (Table S3). It must be noted that four sequences (5–70 strain, 5–75 strain, 5–36 strain and 5–72 strain) were grouped with three different known host strains Mo/CHN-56/2002 strain, Po/E4-14 strain and Hu/1-CHN-01 strain (average nucleotide identity = 85%). Similarly, one sequence (2–24 strain) clustered with Fox/5 strain, Hu/6C2P strain and Un/Florida-12 strain (average nucleotide identity = 90%). This evidence suggests the potential of PBVs for interspecies zoonotic transmission. With regard to the remaining sequences, it was difficult to clearly group them with the unique known host sequences. For example, eight sequences (5–94 strain, 4–52 strain, 5–115 strain, 3-1 strain, 6–7 strain, 1–12 strain, 3–51 strain and 5–77 strain) were clustered into one group with high bootstrap value (1.0) and high average nucleotide identity (95%) and one avian PBV strain Av/AVE 71/2010-3 was in the vicinity of this group of sequences. However, despite a nucleotide identity of 92% and a bootstrap value of 0.24 (< 0.8), this group of strains could not be assigned to the avian PBVs with such weak statistical support. Moreover, some sequences (44 sequences of the 139 sequences) could not be clustered with known sequences; for example, 21 sequences, which were well clustered into one group with a bootstrap value of 0.92 and nucleotide identity of 94%, could not be assigned to any PBV species, because they branched in the deeper part of the phylogenetic tree with no known host strains close to the branches (Fig. 2). Thus, this branch of PBVs might be a new group of PBVs, and more backup data were required to confirm this finding. Although the phylogenetic tree revealed that the PBVs showed wide diversity, the viruses of each host species clustered in distinct clades.

A clade with over 10 sequences and bootstrap values > 0.8 was considered as one group (Fig. 2). Thus, a total of six groups (groups 1–6) were obtained. Except group 4 mentioned earlier, which might be a new group of PBVs with no known host, the other five groups were allocated to known hosts. The porcine PBVs were divided into two groups (groups 1 and 2), because the diversity of the two intergroups was noted to be 35%. The hosts of groups 3, 5 and 6 were avian, monkey and human respectively (Fig. 2). The subsequent evolutionary study was based on these six groups, and the corresponding evolutionary models were as follows: Hasegawa, Kishino, and Yano 1985 (HKY) + Gamma distribution (G) for groups 1 and 2, HKY for group 3, Kimura 1980 (K80) + Invariable Sites (I) for group 4, Transversion Model with equal nucleotide frequencies (TVMef) + I for group 5, and Three-Parameter Model (TPM1) for group 6.

### Assessment of polymorphism

Genetic polymorphism analyses and neutrality tests of the six groups were performed. The results of polymorphism analyses (Table 1) for the six groups showed that the K, S, η and Pa values were the highest (K = 30.582, S = 74, η = 98, Pa = 47) for the monkey strains (group 5) and lowest (K = 6.333, S = 24, η = 24, Pa = 10) for the avian strains (group 3), indicating that monkey strains had a relatively high diversity of nucleic acids, whereas the avian strains had low nucleic acid diversity. The rest of the three groups exhibited similar values. Furthermore, the Ka/Ks value of group 5 was the lowest (0.092125), whereas those of group 3 (0.385676) and group 4 (0.370711) were higher. The sequences of group 5 showed lower levels of non-synonymous substitution, suggesting that despite the high diversity of these sequences with comparatively high polymorphism, synonymous substitution was predominant. Tajima's and D tests were not significant, except for group 4, which presented values of −2.17764 and −1.95013 respectively (Table 1). Moreover, the probability of positive selection of group 4 was higher than that of other groups (Table 2). With regard to group 2, three neutral tests were positive, suggesting that there might be a balancing selection. Overall, except group 4, the PBVs obtained from the treated wastewater samples were noted to prefer a neutral choice.

**Table 1:** Polymorphism analyses and neutrality tests of six groups

	Seq	Hp	K	S	η	Pa	η(s)	Ka	Ks	Ka/Ks	θ^a^	π^b^	Tajima's	D^d^	F^d^	Host
Group 1	14	13	10.604	39	43	18	24	0.036849	0.216865	0.178854	12.578^(3.955)^	0.06321^(0.01264)^	−0.94103	−0.918755	−1.18411	Porcine
Group 2	18	17	18.353	52	61	41	17	0.045231	0.754099	0.146385	15.677^(4.637)^	0.11056^(0.01606)^	0.14454	0.16258	0.18269	Porcine
Group 3	12	11	6.333	24	24	10	14	0.019783	0.11238	0.385676	7.285^(2.412)^	0.03598^(0.00552)^	−0.90266	−0.96741	−1.08234	Avian
Group 4	21	20	10.152	64	85	21	64	0.05963	0.113056	0.370711	21.680^(6.026)^	0.05835^(0.01915)^	−2.17764^d^	−1.95013^c^	−1.98344	Uncultured
Group 5	11	11	30.582	74	98	47	41	0.091158	0.807	0.092125	25.265^(8.626)^	0.16896^(0.01711)^	−0.41194	−0.18922	−0.28001	Monkey
Group 6	12	12	11.455	53	56	11	45	0.034495	0.275658	0.180213	17.882^(5.921)^	0.06508^(0.02254)^	−1.7603	−2.01308	−2.21957	Human

a Variance of θ (free recombination).

b Standard deviation of π.

c Statistical significance *P* < 0.02 (for Fu and Li's test), *P* < 0.01 (for Tajima's test).

d Statistical significance *P* < 0.05 (for all tests).

Hp, haplotypes; K, average number of pairwise difference; S, number of polymorphic (segregating) sites; η, total number of mutations; Pa, parsimony informative sites; η(s), number of singletons; Ka, rate of non-synonymous substitutions; Ks, rate of synonymous substitutions; π, nucleotide diversity; θ, Watterson's mutation parameter (per sequence calculated from S).

**Table 2:** Positive sites analysis for six groups using SLAC, FEL and REL methods

	SLAC	FEL	REL	Host
Group 1	–	–	–	Porcine
Group 2	–	–	–	Porcine
Group 3	–	–	–	Avian
Group 4	–	–	2	Uncultured
Group 5	–	–	1	Monkey
Group 6	–	–	–	Human

### Measurement of selection pressure

The global ω value (0.64) was < 1.0 for all the six groups, which indicated that positive selection was not detected on the RdRp gene in the evolutionary processes of PBVs. Further site-by-site analyses of the six groups were conducted by employing single likelihood ancestor counting (SLAC), fixed effects likelihood (FEL) and random effects likelihood (REL) methods with hyphy package. The results of the SLAC and FEL methods (Table 2) did not reveal any positive selection sites in all the groups. However, the results of the REL method were slightly different, indicating that there were two and one positive selection sites in group 4 (sites 8 and 39) and group 5 (site 36) respectively. Overall, it was concluded that the PBVs preferred neutral selection, which was consistent with the above-mentioned result.

### Substitution rate estimates and MCC tree construct

**Fig 3:**
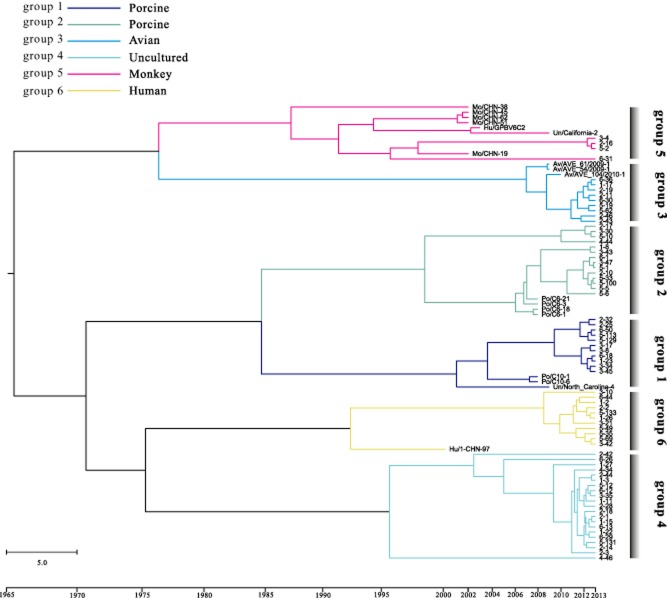
Bayesian maximum credibility (MCC) tree construction based on the sequences of six groups. The branches of different groups were coloured on the basis of different hosts, and at the bottom of the MCC tree represents the years before the last sampling time (2013). Bar, 5 years. The scale on the upper left of the MCC tree showed the different colours of different groups and hosts.

The mean and 95% lower and upper highest posterior density (HPD) were estimated for the nucleic acid substitution rates for all the groups by using the Markov chain Monte Carlo (MCMC) approach. The results (Table 3) showed that the substitution rates estimated for the PBVs ranged from 4.05E-3 to 1.41E-2 subs per site per year. The substitution rates of PBVs were substantially similar in human strains (1.31E-2 subs per site per year), porcine strains (1.41E-2 and 1.21E-2 subs per site per year), and avian strains (1.33E-2 subs per site per year), whereas that of the monkey PBVs was 6.54E-3 subs per site per year, suggesting that PBVs evolve more slowly in monkey hosts than in human, porcine and avian hosts. This difference in the evolutionary rates of PBVs in different hosts might be owing to the different ecological pressures such as the host immune defence. The MCC phylogenetic trees for each group are shown in Fig. 3 with different groups marked with different colours. Interestingly, from the MCC tree, it could be deduced that monkey was the earliest host for PBVs, although the nucleic acid substitution rate for monkey PBVs was found to be relatively low. Furthermore, among the four hosts (monkey, pig, avian, human), the nearest host was avian. However, PBVs from monkey and avian hosts were likely to be from the same ancestor. As expected, the porcine host of groups 1 and 2 clustered. Based on the MCC tree construct, the undetermined group 4 was speculated be the human PBVs.

**Table 3:** Substitution rate for each group of picobirnaviruses

	Mean substitution rate (10^−2^)	Uncorrelated relaxed clock model	Substitution rate HPD (10^−2^)	Host
Group 1	1.41	Lognormal	0.78–2.01	Porcine
Group 2	1.21	Lognormal	0.91–1.46	Porcine
Group 3	1.33	Exponential	0.76–2.05	Avian
Group 4	0.41	Exponential	0.18–0.62	Uncultured
Group 5	0.65	Exponential	0.31–1.04	Monkey
Group 6	1.31	Exponential	0.61–1.98	Human

## Discussion

In the present study, the PBV population in wastewater treatment plant in Changsha Hunan province, China, was investigated. The results obtained showed that the PBVs could be detected in 100% of the samples collected (Fig. 1). Furthermore, the findings indicated that PBVs were extremely stable and resistant to treatment, and thus might be a good indicator of faecal pollution. Wastewater is a huge mixture matrix, containing various viruses disseminated from different hosts (Cantalupo *et al*., 2011). The viruses collected from wastewater often show vast diversity. In the present study, molecular analysis of the PBVs isolated from the collected wastewater samples revealed their high genetic diversity, with all the sequences exhibiting nucleotide identities ranging between 42% and 100%, which is consistent with the findings reported in a previous study of sewage samples from the USA (Symonds *et al*., 2009). The present study is the first to examine the PBVs from a wastewater treatment plant in China, and a total of 139 sequences of PBVs have been submitted to GenBank (Accession Nos. KJ135791 to KJ135929), thus providing more PBV sequences for further studies. In addition, several important conclusions could be drawn from the phylogenetic analysis of the PBV sequences. First, the PBV population in the wastewater treatment plant in China might have originated from monkey, porcine, avian and human hosts, perhaps owing to the extreme stability and resistance of PBVs to treatment, thus making them ubiquitous with the ability to infect different host species in the same setting. Second, the PBVs exhibited interspecies transmission; for example, four strains (5–70 strain, 5–75 strain, 5–36 strain and 5–72 strain) were allocated to three different known host strains, Mo/CHN-56/2002 strain, Po/E4-14 strain and Hu/1-CHN-01 strain (average nucleotide identity = 85%) (Fig. 2), and one strain (2–24 strain) clustered with Fox/5 strain, Hu/6C2P strain and Un/Florida-12 strain (average nucleotide identity = 90%). These pieces of evidence indicated the potential of PBVs for interspecies zoonotic transmission. Similarly, previous studies on simian genotype I PBVs and PBVs in sewage samples from the USA also suggested that PBVs had the potential for interspecies transmission (Ganesh *et al*., 2011). Third, the PBVs presented higher sequence diversity than that expected. Only 53 strains of the 139 sequences were assigned to the host, whereas the host of the remaining 86 strains could not be determined, indicating that numerous PBVs are yet to be detected. Fourth, a new group of PBVs, including 21 sequences (group 4), was identified, which clustered well into one group with a bootstrap value of 0.92 and a nucleotide identity of 94%; however, these PBVs could not be assigned to any known PBV species (Fig. 2).

Evolutionary analysis revealed that the two porcine strains (groups 1 and 2), avian strains (group 3) and human strains (group 6) presented similar results of site-by-site analyses (Table 2) and nucleotide substitution rates (Table 3). Furthermore, it is worth noting that the monkey strains (group 5) exhibited the highest polymorphism (K = 30.582, S = 74, η = 98, Pa = 47) and comparatively lower nucleotide substitution rate (6.54E-3 subs per site per year). The reason for this seemingly contradictory result may be that the monkey strains appeared earlier than the other hosts in the MCC tree (Fig. 3). Hence, it can be concluded that the relationship between the PBVs and the monkey host is very important and that a balance may exist between them. The new PBV group identified (group 4) showed two positive selection sites (sites 8 and site 39), the lowest evolutionary rate (4.05E-3 subs per site per year) and unique characteristics in the neutrality test. The results of Tajima's and F tests were not significant for all the analysed groups, except for group 4, which showed significant (*P* < 0.05) and highly significant (*P* < 0.02) results respectively (Table 1). Similar to most of the recent works, in the present study, a short segment of RdRp gene was used to construct the phylogenetic tree of PBVs and investigate its evolutionary history and phylodynamics. However, the use of one gene as a criterion, particularly, a part of the RdRp gene that is relatively highly conserved, to elucidate the global understanding of one virus is insufficient. Hence, future PBV studies should focus on full-length (segments 1 and 2) sequence analysis to gain a better understanding of the relationship between evolution and pathogenicity of PBVs.

## Experimental procedures

### Collection and concentration of the wastewater samples

Six samples were collected at different points from the stream of a wastewater treatment plant in Changsha, Hunan province, China, in autumn 2012. The recovery of viral particles and nucleic acid extraction was carried out by employing methods previously described (Pina *et al*., 1998). Briefly, 48 ml of sewage sample were ultracentrifuged at 200 000 × *g* for 1 h at 4°C. The sediment was dissolved in 4.8 ml of 0.25 N glycine buffer (pH 9.5) on ice for 30 min, and then the mixture was ultracentrifuged at 10 000 × *g* for 15 min and the suspended solids were removed. The supernatant was ultracentrifuged at 200 000 × *g* for 1 h at 4°C, and the virus in the pellet was resuspended in 0.1 ml of PBS and stored at −80°C.

### Nucleic acid extraction and reverse transcription

The QIAamp MinElute Virus Spin Kit was used for the extraction of nucleic acids. Briefly, 200 μl of concentrated virus and 200 μl of Buffer AL were added into a 1.5 ml tube at 56°C for 15 s. Then, the tube was centrifuged briefly to remove the drops, and the lysate was carefully applied onto the QIAamp MinElute column and centrifuged at 6000 × *g* for 1 min. Subsequently, 500 μl of Buffer AW1, Buffer AW2 and ethanol were respectively added to the mixture and centrifuged at 6000 × *g* for 1 min. Finally, 60 μl of RNase-free water were added to the centre of the membrane. Immediately following nucleic acid extraction, cDNA was synthesized from the extracted RNA by using a First-Strand Synthesis Superscript III Reverse Transcription Kit (Invitrogen, Carlsbad, CA) and stored at −20°C.

### PCR and sequencing of PBVs

A part of the genomic segment 2 of PBVs (genotype I) was amplified by PCR. The specific primers used were PicoB25 TGGTGTGGATGTTTC and PicoB43 ARTGYTGGT CGAACTT (∼ 200 bp) (Martínez *et al*., 2003). The PCR conditions were as follows: initial denaturation at 94°C for 2 min, followed by 40 cycles at 94°C for 1 min, 49°C for 2 min and 72°C for 3 min, and a final elongation step at 72°C for 5 min and 72°C for 10 min. All the PCR products were visualized by agarose gel (2%) electrophoresis with ethidium bromide under UV light. The EasyPure PCR Purification Kit (TransGen, Beijing, China) was used to purify the PCR products. To understand the diversity of the PBVs isolated from the treated wastewater, the positive PCR products from the six samples were cloned into pMD-19 T-vector (Takara, Dalian, China), and the transformants were screened for inserts by PCR and sequenced by Sangon (Sangon, Beijing, China). All the sequences were trimmed by EditSeq of dnaman version 6.0.40. The identities of the positive PCR products were confirmed by comparing the sequences against the GenBank non-redundant database using blastn.

### Phylogenetic analysis

To gain a deeper understanding of the diversity of genotype I PBVs detected in the treated wastewater samples, phylogenetic analysis of the cloned PBVs was executed. The top three similar known sequences from NCBI corresponding to every sequence obtained in the present study were chosen as the reference sequences. All the sequences were aligned and a phylogenetic tree was constructed by using mega 5.0 (Tamura *et al*., 2011). Briefly, clustal of mega 5.0 was used for multiple alignments of all the sequences. A neighbour-joining (NJ) phylogenetic tree was constructed using a Kimura 2-parameter model by mega 5.0. The Kimura 2-parameter model was chosen because the average pairwise Kimura 2-parameter distance was < 1.0 (0.474), which is appropriate for creating an NJ tree. The phylogenetic tree was rooted with the human PBV strain 4-GA-91 (AF246940), which is the prototype strain for genotype II. Bootstrap analysis was performed with 1000 replicates. The bootstrap values > 80% were depicted at the nodes of the tree and non-significant values were omitted. The best-fit model of nucleotide substitution was selected by using jmodeltest 0.1.1 based on AIC (Posada, 2008), and the corresponding evolutionary models were generated: HKY + G for groups 1 and 2, HKY for group 3, K80 + I for group 4, TVMef + I for group 5 and TPM1 for group 6.

### Polymorphism analyses

DNA polymorphism analyses are helpful to understand the evolutionary process, which include determination of the number of haplotypes (Hp), average number of pairwise nucleotide differences within the population (K), number of segregating sites (S) (Watterson, 1975), total number of mutations (Eta), parsimony informative sites (Pa), number of singletons [η(s)], and synonymous and non-synonymous substitution rates (Ka/Ks). The neutrality tests employed involve Tajima's test (Tajima, 1989) and Fu and Li's D and F tests (Fu and Li, 1993). The dnasp 5.0 package (Librado and Rozas, 2009) was used to analyse the results of genetic polymorphism and neutrality tests.

### Analyses of selection pressure

Global ω can reflect the direction and strength of selection. In the present study, the global ω was calculated for all the six groups by using hyphy 2.1.0 (Pond and Muse, 2005). Furthermore, three algorithms, namely the SLAC, FEL and REL methods, were employed to analyse site-specific positive selection pressure for each group by using the same software (Wei *et al*., 2013). The results of SLAC and FEL were deemed to be significant if *P* < 0.05, whereas for REL, Bayes factor > 20 was considered to be significant.

### Analyses of evolutionary rate and construction of MCC tree

The analyses of evolutionary rate can reflect some ecological pressures to a certain extent. In the present study, Bayesian analysis, using the Bayesian MCMC approach, was used to estimate the rate of molecular evolution by employing beast 1.7.2 (Drummond and Rambaut, 2007). For each group, a Bayes factor test was performed using Tracer v1.5 to choose the uncorrelated lognormal (UCLD) or exponential (UCED) distributions of strict and relaxed molecular clock. The strict clock assumes a single evolutionary rate along all branches, and the UCLD and UCED clocks allow evolutionary rates to vary along branches within lognormal and exponential distributions respectively. The UCLD clock was found to be the most appropriate model for groups 1 and 2. With regard to the other groups, the UCED was noted to be the best-fit clock model. The uncertainty was addressed as 95% HPD intervals. Subsequently, MCC tree was constructed by using treeannotator 1.7.1 after discarding burn-in of 15%. The MCC tree was visualized with figtree 1.3.1 and coreldraw x5.

### Nucleotide sequence accession numbers

All the sequences determined in this study were deposited in GenBank under accession numbers KJ135791 to KJ135929.

## Supporting Information

**Table S1.** Accession numbers of the 139 sequences we obtained used in this study. **Table S2.** Accession numbers of the 65 reference sequences from NCBI used in this study. **Table S3.** The hosts of the sequences we obtained in this study.
