# Supplementary material for: Detection and evolutionary analysis of picobirnaviruses in treated wastewater
Source: Microb Biotechnol. 2014 Dec 27;8(3):474–82. doi: 10.1111/1751-7915.12239 (PMC4408179; doi:10.1111/1751-7915.12239)
Supplement: Supplementary file 1 [file mbt20008-0474-sd1.doc]

**Table S1** Accession numbers of the 139 sequences we obtained used in this study.

| Numbers | Strains | Source | Accession Number |
| --- | --- | --- | --- |
| 1 | 1-1 | sample1 number1 | KJ135791 |
| 2 | 1-2 | sample1 number2 | KJ135792 |
| 3 | 1-3 | sample1 number3 | KJ135793 |
| 4 | 1-8 | sample1 number8 | KJ135794 |
| 5 | 1-11 | sample1 number11 | KJ135795 |
| 6 | 1-12 | sample1 number12 | KJ135796 |
| 7 | 1-14 | sample1 number14 | KJ135797 |
| 8 | 1-15 | sample1 number15 | KJ135798 |
| 9 | 1-17 | sample1 number17 | KJ135799 |
| 10 | 1-21 | sample1 number21 | KJ135800 |
| 11 | 1-22 | sample1 number22 | KJ135801 |
| 12 | 1-23 | sample1 number23 | KJ135802 |
| 13 | 1-25 | sample1 number25 | KJ135803 |
| 14 | 1-26 | sample1 number26 | KJ135804 |
| 15 | 1-27 | sample1 number27 | KJ135805 |
| 16 | 2-1 | sample2 number1 | KJ135806 |
| 17 | 2-2 | sample2 number2 | KJ135807 |
| 18 | 2-3 | sample2 number3 | KJ135808 |
| 19 | 2-4 | sample2 number4 | KJ135809 |
| 20 | 2-5 | sample2 number5 | KJ135810 |
| 21 | 2-6 | sample2 number6 | KJ135811 |
| 22 | 2-8 | sample2 number8 | KJ135812 |
| 23 | 2-10 | sample2 number10 | KJ135813 |
| 24 | 2-11 | sample2 number11 | KJ135814 |
| 25 | 2-12 | sample2 number12 | KJ135815 |
| 26 | 2-14 | sample2 number14 | KJ135816 |
| 27 | 2-16 | sample2 number16 | KJ135817 |
| 28 | 2-17 | sample2 number17 | KJ135818 |
| 29 | 2-18 | sample2 number18 | KJ135819 |
| 30 | 2-19 | sample2 number19 | KJ135820 |
| 31 | 2-20 | sample2 number20 | KJ135821 |
| 32 | 2-22 | sample2 number22 | KJ135822 |
| 33 | 2-24 | sample2 number24 | KJ135823 |
| 34 | 2-25 | sample2 number25 | KJ135824 |
| 35 | 2-27 | sample2 number27 | KJ135825 |
| 36 | 2-28 | sample2 number28 | KJ135826 |
| 37 | 2-32 | sample2 number32 | KJ135827 |
| 38 | 2-36 | sample2 number36 | KJ135828 |
| 39 | 2-39 | sample2 number39 | KJ135829 |
| 40 | 2-42 | sample2 number42 | KJ135830 |
| 41 | 2-43 | sample2 number43 | KJ135831 |
| 42 | 2-44 | sample2 number44 | KJ135832 |
| 43 | 2-46 | sample2 number46 | KJ135833 |
| 44 | 2-50 | sample2 number50 | KJ135834 |
| 45 | 3-1 | sample3 number1 | KJ135835 |
| 46 | 3-2 | sample3 number2 | KJ135836 |
| 47 | 3-4 | sample3 number4 | KJ135837 |
| 48 | 3-8 | sample3 number8 | KJ135838 |
| 49 | 3-9 | sample3 number9 | KJ135839 |
| 50 | 3-10 | sample3 number10 | KJ135840 |
| 51 | 3-12 | sample3 number12 | KJ135841 |
| 52 | 3-15 | sample3 number15 | KJ135842 |
| 53 | 3-17 | sample3 number17 | KJ135843 |
| 54 | 3-19 | sample3 number19 | KJ135844 |
| 55 | 3-21 | sample3 number21 | KJ135845 |
| 56 | 3-29 | sample3 number29 | KJ135846 |
| 57 | 3-30 | sample3 number30 | KJ135847 |
| 58 | 3-31 | sample3 number31 | KJ135848 |
| 59 | 3-33 | sample3 number33 | KJ135849 |
| 60 | 3-34 | sample3 number34 | KJ135850 |
| 61 | 3-35 | sample3 number35 | KJ135851 |
| 62 | 3-41 | sample3 number41 | KJ135852 |
| 63 | 3-42 | sample3 number42 | KJ135853 |
| 64 | 3-43 | sample3 number43 | KJ135854 |
| 65 | 3-45 | sample3 number45 | KJ135855 |
| 66 | 3-47 | sample3 number47 | KJ135856 |
| 67 | 3-50 | sample3 number50 | KJ135857 |
| 68 | 3-51 | sample3 number51 | KJ135858 |
| 69 | 4-5 | sample4 number5 | KJ135859 |
| 70 | 4-6 | sample4 number6 | KJ135860 |
| 71 | 4-31 | sample4 number31 | KJ135861 |
| 72 | 4-32 | sample4 number32 | KJ135862 |
| 73 | 4-34 | sample4 number34 | KJ135863 |
| 74 | 4-37 | sample4 number37 | KJ135864 |
| 75 | 4-44 | sample4 number44 | KJ135865 |
| 76 | 4-46 | sample4 number46 | KJ135866 |
| 77 | 4-48 | sample4 number48 | KJ135867 |
| 78 | 4-49 | sample4 number49 | KJ135868 |
| 79 | 4-52 | sample4 number52 | KJ135869 |
| 80 | 5-1 | sample5 number1 | KJ135870 |
| 81 | 5-2 | sample5 number2 | KJ135871 |
| 82 | 5-3 | sample5 number3 | KJ135872 |
| 83 | 5-5 | sample5 number5 | KJ135873 |
| 84 | 5-6 | sample5 number6 | KJ135874 |
| 85 | 5-10 | sample5 number10 | KJ135875 |
| 86 | 5-12 | sample5 number12 | KJ135876 |
| 87 | 5-14 | sample5 number14 | KJ135877 |
| 88 | 5-19 | sample5 number19 | KJ135878 |
| 89 | 5-33 | sample5 number33 | KJ135879 |
| 90 | 5-35 | sample5 number35 | KJ135880 |
| 91 | 5-36 | sample5 number36 | KJ135881 |
| 92 | 5-41 | sample5 number41 | KJ135882 |
| 93 | 5-42 | sample5 number42 | KJ135883 |
| 94 | 5-44 | sample5 number44 | KJ135884 |
| 95 | 5-45 | sample5 number45 | KJ135885 |
| 96 | 5-49 | sample5 number49 | KJ135886 |
| 97 | 5-62 | sample5 number62 | KJ135887 |
| 98 | 5-68 | sample5 number68 | KJ135888 |
| 99 | 5-69 | sample5 number69 | KJ135889 |
| 100 | 5-70 | sample5 number70 | KJ135890 |
| 101 | 5-72 | sample5 number72 | KJ135891 |
| 102 | 5-74 | sample5 number74 | KJ135892 |
| 103 | 5-75 | sample5 number75 | KJ135893 |
| 104 | 5-77 | sample5 number77 | KJ135894 |
| 105 | 5-78 | sample5 number78 | KJ135895 |
| 106 | 5-88 | sample5 number88 | KJ135896 |
| 107 | 5-89 | sample5 number89 | KJ135897 |
| 108 | 5-92 | sample5 number92 | KJ135898 |
| 109 | 5-94 | sample5 number94 | KJ135899 |
| 110 | 5-95 | sample5 number95 | KJ135900 |
| 111 | 5-100 | sample5 number100 | KJ135901 |
| 112 | 5-108 | sample5 number108 | KJ135902 |
| 113 | 5-111 | sample5 number111 | KJ135903 |
| 114 | 5-113 | sample5 number113 | KJ135904 |
| 115 | 5-115 | sample5 number115 | KJ135905 |
| 116 | 5-126 | sample5 number126 | KJ135906 |
| 117 | 5-129 | sample5 number129 | KJ135907 |
| 118 | 5-131 | sample5 number131 | KJ135908 |
| 119 | 5-133 | sample5 number133 | KJ135909 |
| 120 | 6-1 | sample6 number1 | KJ135910 |
| 121 | 6-4 | sample6 number4 | KJ135911 |
| 122 | 6-6 | sample6 number6 | KJ135912 |
| 123 | 6-7 | sample6 number7 | KJ135913 |
| 124 | 6-8 | sample6 number8 | KJ135914 |
| 125 | 6-9 | sample6 number9 | KJ135915 |
| 126 | 6-11 | sample6 number11 | KJ135916 |
| 127 | 6-12 | sample6 number12 | KJ135917 |
| 128 | 6-13 | sample6 number13 | KJ135918 |
| 129 | 6-18 | sample6 number18 | KJ135919 |
| 130 | 6-23 | sample6 number23 | KJ135920 |
| 131 | 6-26 | sample6 number26 | KJ135921 |
| 132 | 6-28 | sample6 number28 | KJ135922 |
| 133 | 6-29 | sample6 number29 | KJ135923 |
| 134 | 6-30 | sample6 number30 | KJ135924 |
| 135 | 6-31 | sample6 number31 | KJ135925 |
| 136 | 6-36 | sample6 number36 | KJ135926 |
| 137 | 6-44 | sample6 number44 | KJ135927 |
| 138 | 6-46 | sample6 number46 | KJ135928 |
| 139 | 6-50 | sample6 number50 | KJ135929 |

**Table S2** Accession numbers of the 65 reference sequences from NCBI used in this study.

| Numbers | Strains | Abbreviation | Accession Number |
| --- | --- | --- | --- |
| 140 | Human picobirnavirus strain 1-CHN-97 | Hu/1-CHN-97 | AF246939 |
| 141 | Human picobirnavirus strain 1-CHN-01 | Hu/1-CHN-01 | AJ504794 |
| 142 | Human picobirnavirus strain GPBV6C2P | Hu/6C2P | AB526254 |
| 143 | Human picobirnavirus strain GPBV9 | Hu/GPBV9 | AB517735 |
| 144 | Human picobirnavirus strain GPBV6C4 | Hu/GPBV6C4 | AB517734 |
| 145 | Human picobirnavirus strain GPBV6C2 | Hu/GPBV6C2 | AB517732 |
| 146 | Human picobirnavirus strain 4-GA-91 | Hu/4-GA-91 | AF246940 |
| 147 | Porcine picobirnavirus strain C10 clone 1 | Po/C10-1 | AM706363 |
| 148 | Porcine picobirnavirus strain C10 clone 6 | Po/C10-6 | AM706365 |
| 149 | Porcine picobirnavirus strain C10 clone 14 | Po/C10-14 | AM706366 |
| 150 | Porcine picobirnavirus strain C6 clone 1 | Po/C6-1 | AM706357 |
| 151 | Porcine picobirnavirus strain C6 clone 3 | Po/C6-3 | AM706358 |
| 152 | Porcine picobirnavirus strain C6 clone 18 | Po/C6-18 | AM706361 |
| 153 | Porcine picobirnavirus strain C6 clone 21 | Po/C6-21 | AM706362 |
| 154 | Porcine picobirnavirus strain E4 clone 14 | Po/E4-14 | AM706397 |
| 155 | Porcine picobirnavirus strain D4 clone 1 | Po/D4-1 | AM706367 |
| 156 | Picobirnavirus monkey/CHN-17/2002 | Mo/CHN-17/2002 | JQ710494 |
| 157 | Picobirnavirus monkey/CHN-27/2002 | Mo/CHN-27/2002 | JQ710480 |
| 158 | Picobirnavirus monkey/CHN-60/2002 | Mo/CHN-60/2002 | JQ710500 |
| 159 | Picobirnavirus monkey/CHN-48/2002 | Mo/CHN-48/2002 | JQ710484 |
| 160 | Picobirnavirus monkey/CHN-46/2002 | Mo/CHN-46/2002 | JQ710483 |
| 161 | Picobirnavirus monkey/CHN-79/2003 | Mo/CHN-79/2003 | JQ710505 |
| 162 | Picobirnavirus monkey/CHN-3/2002 | Mo/CHN-3/2002 | JQ710490 |
| 163 | Picobirnavirus monkey/CHN-61/2002 | Mo/CHN-61/2002 | JQ710501 |
| 164 | Picobirnavirus monkey/CHN-56/2002 | Mo/CHN-56/2002 | JQ710499 |
| 165 | Picobirnavirus monkey/CHN-33/2002 | Mo/CHN-33/2002 | JQ710481 |
| 166 | Picobirnavirus monkey/CHN-34/2002 | Mo/CHN-34/2002 | JQ710469 |
| 167 | Picobirnavirus monkey/CHN-19/2002 | Mo/CHN-19/2002 | JQ710495 |
| 168 | Picobirnavirus monkey/CHN-38/2003 | Mo/CHN-38/2002 | JQ710482 |
| 169 | Picobirnavirus monkey/CHN-62/2002 | Mo/CHN-62/2002 | JQ710473 |
| 170 | Picobirnavirus monkey/CHN-51/2002 | Mo/CHN-51/2002 | JQ710485 |
| 171 | Picobirnavirus monkey/CHN-45/2002 | Mo/CHN-45/2002 | JQ710471 |
| 172 | Avian picobirnavirus strainAVE-108/2011 clone 1 | Av/AVE 108/2011-1 | KC865829 |
| 173 | Avian picobirnavirus strainAVE-104/2010 clone 3 | Av/AVE 104/2010-3 | KC865826 |
| 174 | Avian picobirnavirus strainAVE-104/2010 clone 1 | Av/AVE 104/2010-1 | KC865824 |
| 175 | Avian picobirnavirus strainAVE-71/2010 clone 3 | Av/AVE 71/2010-3 | KC865817 |
| 176 | Avian picobirnavirus strainAVE-101/2010 clone 2 | Av/AVE 101/2010-2 | KC865823 |
| 177 | Avian picobirnavirus strainAVE-77/2010 clone 3 | Av/AVE 77/2010-3 | KC865821 |
| 178 | Avian picobirnavirus strainAVE-70/2010 clone 1 | Av/AVE 70/2010-1 | KC865811 |
| 179 | Avian picobirnavirus strainAVE-61/2010 clone 1 | Av/AVE 61/2009-1 | KC865807 |
| 180 | Avian picobirnavirus strainAVE-54/2010 clone 1 | Av/AVE 61/2009-2 | KC865802 |
| 181 | Avian picobirnavirus strainAVE-48/2010 clone 2 | Av/AVE 48/2009-2 | KC865800 |
| 182 | Avian picobirnavirus strainAVE-42/2010 clone 1 | Av/AVE 42/2009-1 | KC865798 |
| 183 | Fox picobirnavirus isolate Fox/5 | Fox/5 | KC692366 |
| 184 | Uncultured picobirnavirus clone Washington Raw Sewage13 | Un/Washington-13 | EU938911 |
| 185 | Uncultured picobirnavirus clone Washington Raw Sewage10 | Un/Washington-10 | EU938908 |
| 186 | Uncultured picobirnavirus clone Louisiana Raw Sewage13 | Un/Louisiana-13 | EU938822 |
| 187 | Uncultured picobirnavirus clone Louisiana Raw Sewage1 | Un/Louisiana-1 | EU938810 |
| 188 | Uncultured picobirnavirus clone Maryland Raw Sewage18 | Un/Maryland-18 | EU938860 |
| 189 | Uncultured picobirnavirus clone Maryland Raw Sewage9 | Un/Maryland-9 | EU938851 |
| 190 | Uncultured picobirnavirus clone Maryland Raw Sewage14 | Un/Maryland-14 | EU938856 |
| 191 | Uncultured picobirnavirus clone Maryland Raw Sewage17 | Un/Maryland-17 | EU938859 |
| 192 | Uncultured picobirnavirus clone Florida Raw Sewage25 | Un/Florida-25 | EU938796 |
| 193 | Uncultured picobirnavirus clone Florida Raw Sewage12 | Un/Florida-12 | EU938783 |
| 194 | Uncultured picobirnavirus clone Oregon Raw Sewage13 | Un/Oregon-13 | EU938896 |
| 195 | Uncultured picobirnavirus clone Oregon Raw Sewage7 | Un/Oregon-7 | EU938890 |
| 196 | Uncultured picobirnavirus clone Oregon Raw Sewage2 | Un/Oregon-2 | EU938885 |
| 197 | Uncultured picobirnavirus clone Oregon Raw Sewage8 | Un/Oregon-8 | EU938891 |
| 198 | Uncultured picobirnavirus clone Alabama Raw Sewage11 | Un/Alabama-11 | EU938717 |
| 199 | Uncultured picobirnavirus clone North Carolina Raw Sewage10 | Un/North Carolina-10 | EU938871 |
| 200 | Uncultured picobirnavirus clone North Carolina Raw Sewage4 | Un/North Carolina-4 | EU938865 |
| 201 | Uncultured picobirnavirus clone California Raw Sewage2 | Un/California-2 | EU938723 |
| 202 | Uncultured picobirnavirus clone California Raw Sewage6 | Un/California-6 | EU938727 |
| 203 | Uncultured picobirnavirus clone California Raw Sewage13 | Un/California-13 | EU938734 |
| 204 | Uncultured picobirnavirus clone New Jersey Raw Sewage6 | Un/New Jersey-6 | EU938877 |

**Table S3** The hosts of the sequences we obtained in this study.

| Strains | Accession Number | host | Strains | Accession Number | host |
| --- | --- | --- | --- | --- | --- |
| 1-1 | KJ135791 | porcine | 1-2 | KJ135792 | human |
| 1-8 | KJ135794 |  | 1-26 | KJ135804 |  |
| 1-23 | KJ135802 |  | 2-2 | KJ135807 |  |
| 2-10 | KJ135813 |  | 3-10 | KJ135840 |  |
| 2-17 | KJ135818 |  | 3-21 | KJ135845 |  |
| 2-25 | KJ135824 |  | 3-42 | KJ135853 |  |
| 2-32 | KJ135827 |  | 5-35 | KJ135880 |  |
| 3-8 | KJ135838 |  | 5-49 | KJ135886 |  |
| 3-17 | KJ135843 |  | 5-69 | KJ135889 |  |
| 3-30 | KJ135847 |  | 5-133 | KJ135909 |  |
| 3-34 | KJ135850 |  | 2-16 | KJ135817 | monkey |
| 3-43 | KJ135854 |  | 3-4 | KJ135837 |  |
| 3-45 | KJ135855 |  | 5-2 | KJ135871 |  |
| 3-47 | KJ135856 |  | 6-31 | KJ135925 |  |
| 4-44 | KJ135865 |  | 1-17 | KJ135799 | avian |
| 5-1 | KJ135870 |  | 2-11 | KJ135814 |  |
| 5-5 | KJ135873 |  | 2-19 | KJ135820 |  |
| 5-6 | KJ135874 |  | 2-43 | KJ135831 |  |
| 5-10 | KJ135875 |  | 2-46 | KJ135833 |  |
| 5-33 | KJ135879 |  | 3-31 | KJ135848 |  |
| 5-100 | KJ135901 |  | 3-33 | KJ135849 |  |
| 5-113 | KJ135904 |  | 4-32 | KJ135862 |  |
| 5-129 | KJ135907 |  | 5-19 | KJ135878 |  |
|  |  |  | 5-62 | KJ135887 |  |
|  |  |  | 6-30 | KJ135924 |  |
|  |  |  | 6-36 | KJ135926 |  |
